# BertTCR: a Bert-based deep learning framework for predicting cancer-related immune status based on T cell receptor repertoire

**DOI:** 10.1093/bib/bbae420

**Published:** 2024-08-23

**Authors:** Min Zhang, Qi Cheng, Zhenyu Wei, Jiayu Xu, Shiwei Wu, Nan Xu, Chengkui Zhao, Lei Yu, Weixing Feng

**Affiliations:** College of Intelligent Systems Science and Engineering, Harbin Engineering University, No. 145 Nantong Street, Nangang District, Harbin, 150001, China; College of Intelligent Systems Science and Engineering, Harbin Engineering University, No. 145 Nantong Street, Nangang District, Harbin, 150001, China; College of Intelligent Systems Science and Engineering, Harbin Engineering University, No. 145 Nantong Street, Nangang District, Harbin, 150001, China; College of Intelligent Systems Science and Engineering, Harbin Engineering University, No. 145 Nantong Street, Nangang District, Harbin, 150001, China; College of Intelligent Systems Science and Engineering, Harbin Engineering University, No. 145 Nantong Street, Nangang District, Harbin, 150001, China; Institute of Biomedical Engineering and Technology, Shanghai Engineering Research Center of Molecular Therapeutics and New Drug Development, School of Chemistry and Molecular Engineering, East China Normal University, No. 500 Dongchuan Road, Shanghai, 200241, China; Shanghai Unicar-Therapy Bio-medicine Technology Co., Ltd, No. 1525 Minqiang Road, Shanghai, 201612, China; College of Intelligent Systems Science and Engineering, Harbin Engineering University, No. 145 Nantong Street, Nangang District, Harbin, 150001, China; Shanghai Unicar-Therapy Bio-medicine Technology Co., Ltd, No. 1525 Minqiang Road, Shanghai, 201612, China; Institute of Biomedical Engineering and Technology, Shanghai Engineering Research Center of Molecular Therapeutics and New Drug Development, School of Chemistry and Molecular Engineering, East China Normal University, No. 500 Dongchuan Road, Shanghai, 200241, China; Shanghai Unicar-Therapy Bio-medicine Technology Co., Ltd, No. 1525 Minqiang Road, Shanghai, 201612, China; College of Intelligent Systems Science and Engineering, Harbin Engineering University, No. 145 Nantong Street, Nangang District, Harbin, 150001, China

**Keywords:** T cell receptor, pre-trained protein-BERT model, deep learning, multiple instance learning, cancer-related immune status prediction

## Abstract

The T cell receptor (TCR) repertoire is pivotal to the human immune system, and understanding its nuances can significantly enhance our ability to forecast cancer-related immune responses. However, existing methods often overlook the intra- and inter-sequence interactions of T cell receptors (TCRs), limiting the development of sequence-based cancer-related immune status predictions. To address this challenge, we propose BertTCR, an innovative deep learning framework designed to predict cancer-related immune status using TCRs. BertTCR combines a pre-trained protein large language model with deep learning architectures, enabling it to extract deeper contextual information from TCRs. Compared to three state-of-the-art sequence-based methods, BertTCR improves the AUC on an external validation set for thyroid cancer detection by 21 percentage points. Additionally, this model was trained on over 2000 publicly available TCR libraries covering 17 types of cancer and healthy samples, and it has been validated on multiple public external datasets for its ability to distinguish cancer patients from healthy individuals. Furthermore, BertTCR can accurately classify various cancer types and healthy individuals. Overall, BertTCR is the advancing method for cancer-related immune status forecasting based on TCRs, offering promising potential for a wide range of immune status prediction tasks.

## Introduction

T cells play a vital role in the adaptive immune response against foreign and self-antigens [1–3]. Changes in the T cell receptor (TCR) profile have become an important marker for monitoring the immune status of the body, so it has become critical to accurately evaluate and compare the status of the TCR repertoire [4, 5]. In the T cell receptors (TCRs), the variable region exhibits high diversity [6–10], which is crucial for the TCR’s specific recognition of antigens [11]. Within these variable regions, three areas exhibit particularly significant variations in their amino acid (AA) sequences, forming spatial configurations that are complementary to the antigen epitopes [12, 13]. These three regions (CDR1, CDR2, and CDR3) are known as the complementarity determining regions (CDR) [14]. Notably, the CDR3 region is the most diverse part of the entire variable region and is the key area that directly binds with the antigen during the antigen recognition process [15]. In general, TCR repertoire analysis refers to the study of the CDR3 region. By studying an individual’s intra- and inter-sequence interactions of TCR repertoire, we can obtain information about the immune response and the shared receptors associated with specific cancer to distinguish cancer-related immune status (cancer or health) [16–20]. Therefore, the research on predicting individual immune status based on the TCR repertoire holds important clinical application potential.

The studies have been showed that various predicators play important role to decipher the of important biological various biological molecules including proteins and enzymes [21–25]. Traditional machine learning methods such as support vector machines, random forests, and logistic regression are unable to effectively represent features of multiple protein sequences, handle inconsistent lengths of TCRs, and capture correlations between sequences. These limitations may restrict their ability to capture the complex features of TCRs, thus posing significant challenges in understanding and analyzing individual TCR repertoire, thereby affecting the accuracy of predicting cancer-related immune status. Recently, the introduction of deep learning models has marked a substantial advancement in processing large-scale, high-throughput TCR sequencing data [26–31]. For example, Emerson et al. (2017) proposed a statistical classification framework for predicting the status of cytomegalovirus [32], Yokota’s method (2017) aimed to differentiate TCR repertoire in high-dimensional sequence spaces [33], and the GIANA (2021) utilized isometric transformations for TCR clustering [34]. All three methods focus on the similarity comparison between the whole sequence and fail to pay attention to the CDR3’s short peptide motif of TCR, which is very important for antigen binding specificity. Beshnova’s (2020) deep learning model (DeepCAT), Ostmeyer’s (2019) multiple instance learning method, and Kim’s (2022) Atchley matrix sequence embedding method all overlook the rich contextual information and interrelations among TCRs. Specifically, Ostmeyer’s model predicts individual cancer probabilities using single TCR sequences, yet fails to adequately consider the intra- and inter-sequence interactions of TCRs. Additionally, DeepCAT only covers 5 TCR lengths, failing to utilize features of TCRs with other lengths [35–37]. Although DeepLION (2022) considers the interrelations of TCRs, its TCR embedding method and classifier failed to fully capture the complexity and contextual information of TCRs, greatly limiting its performance [38]. Zhou et al. (2024) and Cai et al. (2024) focus more on using the statistical features of the sequence rather than the characteristics of the CDR3 sequence [39, 40]. These methods not only overlook the critical role of CDR3 short peptide motifs and the complex interactions among TCRs, but also exhibit shortcomings in embedding protein sequences.

To address these limitations of existing methods, we propose a deep learning framework based on self-supervised pre-trained called BertTCR. Compared to traditional approaches, BertTCR demonstrates significant advantages in feature extraction and cancer-related immune status prediction. Our contributions include four aspects: firstly, we utilize pre-trained protein–bidirectional encoder representations from transformers (BERT) models to convert TCRs into high-dimensional representations, thereby eliminating the need for manual feature extraction and effectively enhancing contextual information. Secondly, we design the CME predictor component that combines convolutional neural network (CNN), multiple instance learning (MIL) and ensemble learning (EL), capable of capturing short peptide motifs related to antigen binding and considering the intra- and inter-sequence interactions among TCRs, thereby enhancing the robustness and generalization ability of the model. Thirdly, we propose a novel approach to defining a health score to assess the health status of healthy or patients and provide early intervention for those with slightly lower immune scores. Finally, our architecture is flexibly applied to multi-class tasks for predicting immune status. Through this research, we not only extend previous work on binary cancer prediction but also address two additional complex issues: a universal model for cancer detection and multi-class challenges. Overall, our study makes significant contributions to the early diagnosis and intervention of cancer-related immune status and provides accurate and personalized medical recommendations for the healthcare field.

## Materials and methods

In this study, we propose a deep learning framework named BertTCR, which aims to predict personal immune status through TCRs analysis. Our work mainly includes data preprocessing, embedding of TCRs using a pre-trained protein-BERT model, the construction of CME predictor [41], as well as evaluating model performance using internal test sets and external TCR-seq validation sets.

### Overview of BertTCR

**Figure 1:**
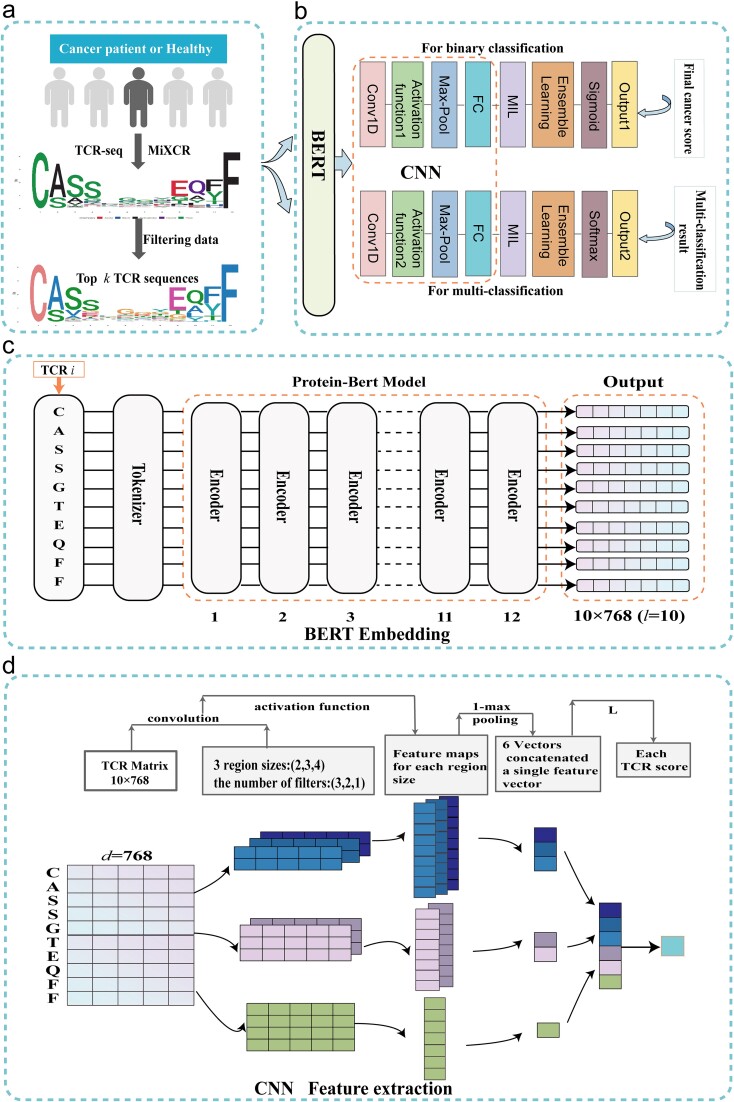
BertTCR for accurate cancer-related immune status prediction. (a) Processing CDR3 sequence data for downstream analysis. Initially, the MiXCR tool was employed to assemble TCRs, generating TCR sequence data that meets the research requirements. The top k CDR3 sequences (CDR3s) with the highest abundance were selected for further analysis. (b) The overall architecture of the model. It provides an overview of the various components and their interactions. (c) The details of embedding TCRs using a pretrained protein-BERT model. For a sequence of length l, the model performs an embedding operation, generating a vector representation of dimensions (l × 768). (d) The details of CNN in BertTCR. The generated TCR sequence matrix is processed through convolutional layers with different scales (kernel sizes of 2, 3, 4) for feature extraction. It is then passed through max pooling and linear layers, ultimately producing a cancer score for the individual sequence.

The workflow is illustrated in Fig. 1. Initially, we employed the MiXCR tool to assemble TCRs, generating TCR sequence data that meets the research requirements [42]. We select the top k CDR3 sequences (CDR3s) with the highest abundance for further analysis, as shown in Fig. 1a. The overall architecture of the model is detailed in Fig. 1b, providing an overview of the various components and their interactions. Next, we use a pre-trained protein-BERT model to embed these chosen sequences, with detailed information provided in Fig. 1c. Then, feature extraction is performed using CNN, and a score is obtained for each sequence, as shown in Fig. 1d. Finally, we utilize MIL and ensemble learning techniques to predict the final cancer score.

### Datasets

The data sources for this study are two publicly available databases: the NCBI database and the Zenodo database. Details about the databases used in this work are described as follows.

**NCBI database.** We used the publicly available TCR-seq data from the National Center for Biotechnology Information (NCBI), which includes 22 groups of peripheral blood mononuclear cell (PBMC) and tumor-infiltrating T lymphocyte (TIL) samples with 17 diverse cancer types and 19 groups of healthy PBMC individuals.

**Zenodo database**. We used the publicly available TCR-seq data from the open-access Zenodo database [43], which contains one group of healthy PBMC individuals.

More detailed information about the databases and their usage can be found in Table S1.

### Data preprocessing

To obtain the CDR3 sequences of TCRβ in the NCBI database, the Sequence Read Archive (SRA) Toolkit is a set of tools developed by the NCBI for processing sequencing data from the SRA. We used the sratoolkit.2.11.3-win64 tool to download SRA data format and converted it to fastq data format. Further processing of these files was performed using the MiXCR tool, which included aligning, assembling, and annotating the highly variable CDR3 regions within the TCRs [44]. Additionally, data from the Zenodo dataset can be downloaded from their website and used directly.

**Determining the length range of CDR3s.** To determine the length range of CDR3s in the study, the frequency distribution of CDR3s lengths was examined using the dataset with bioproject id PRJEB33490 on the NCBI. As shown in Fig. S1, this analysis was conducted in healthy individuals (98 samples), solid tumors (141 samples), and hematological tumors (108 samples). The results indicated that the length distributions of these three categories of sequences approximated a Gaussian distribution, with sequence lengths ranging from 10 to 24 encompassing the majority of sequences in the samples. Specifically, CDR3s with lengths between 10 and 24 accounted for 99.8% of all sequences in healthy individuals, 99.8% in solid tumors, and 98.9% in hematological cancers. What’s more, Stille et al. [45] indicates that CDR3s shorter than nine AA generally do not interact with antigens, while sequences between 12-19 AA are most frequently in contact with antigen epitopes. Our model involves sequence alignment issues, where sequences that are too long or too short can lead to significant errors. Therefore, the sequence length considered in this study ranges from 10 to 24.

**Filtering low-quality CDR3s.** To ensure that model construction is not influenced by low-quality CDR3s, we conducted screening of each sample and only retained CDR3s meeting the following criteria: (i) resolved variable gene loci; (ii) exclusion of special characters (X, *, and _); (iii) Beginning with cysteine (C) and ending with phenylalanine (F) [46]; and (iv) length ranging from 10 to 24. The data analysis after each filtering step, including the proportion of remaining data at each step, the distribution of remaining data, and the diversity of remaining data, is shown in Fig. S2, Fig. S3, and Fig. S4, respectively. After completing data processing, we sorted the retained TCRs in descending order of clone frequency, and extracted the top $k$ sequences for model training.

**Selecting the number of sequences** $k$ . To determine the number of sequences in the model, during the training and testing of each sample, different values of $k$, namely 10, 100, and 1000, were considered. The experimental results indicate that when $k=10$, the performance of the model is not satisfactory, with an AUC value of only 0.671, as shown in Fig. S5. However, when $k=100$, there is a significant improvement in the model performance, with the highest AUC value reaching 0.99. To prevent overfitting, the option of $k=1000$ was also excluded, and finally, $k$ is set to 100.

### BERT embedding

We utilize the neural network in the TAPE library to embed TCRs, which is based on the pre-trained BERT. The TAPE model is developed through a self-supervised learning approach, utilizing a large-scale dataset of more than 31 million protein sequences.

In order to capture the relationships and contextual information between CDR3s of TCRs and enhance the representation capacity of features, we utilized a pre-trained protein-BERT model to embed CDR3s [47–49]. During the embedding process, each AA was encoded as a token. The input protein sequence was embedded as a token sequence. The pre-trained protein-BERT model in TAPE consisted of 12 self-attention layers, with each layer containing 12 attention heads. This architecture enabled the model to capture the complex relationships and dependencies within the input AA sequence. For a given sequence of length L, the output of this model was a $L\times 768$ dimensional vector. These vectors encoded rich contextual information for each position in the CDR3 sequence, thus facilitating downstream prediction tasks.

When using self-attention mechanisms to calculate the correlation between each AA in the TCRs [50, 51], first prepare an input sequence $x=\left({x}_1,{x}_2,...,{x}_L\right)$,where each element ${x}_i$ represents an AA. Query vector (Q), key vector (K), and value vector (V) are created through linear transformations, which can be achieved by multiplying the sequence by the corresponding weight matrix. Then, to stabilize the gradient, the scores are scaled by dividing by the square root of ${d}_k$, and the $score\left({x}_i,{x}_j\right)$ is calculated for each pair of AAs $\left({x}_i,{x}_j\right)$ in the sequence, as formula (1). Next, the scores for each AA are normalized using the softmax function, which exponentiates the scores and divides them by the sum of all scores. The normalized scores are used as weights to multiply each AA by its corresponding weight, and they are then weighted and summed [52]. Through this calculation, a weighted representation of each AA ${x}_i$ with other AAs ${x}_j$ is obtained, which is used to capture important information in the TCR sequence. The matrix form operation of the self-attention layer is as follows:


(1) \begin{equation*} score\left({x}_i,{x}_j\right)=\frac{Q{K}^T}{\sqrt{d_k}} \end{equation*}

(2) \begin{equation*} A\left(Q,K,V\right)= soft\max \left(\frac{Q{K}^T}{\sqrt{d_k}}\right)V \end{equation*}

where Q is the query vector, K is the key vector, and dk is the dimension of the key vector (64 dimensions). In addition, by using multiple layers of self-attention mechanisms, the original input sequence can be transformed into a deeper representation. In the entire model, the outputs of 12 self-attention heads are used and concatenated to provide a final continuous vector of $L\times 768$ dimensions for each TCR sequence and $100\times L\times 768$ dimensional vector for each sample.

We also experimented with a protein-BERT model that was fine-tuned using TCRs, but the original protein-BERT model had the best performance.

### CME predictor

#### CNN extracts short peptide motifs of various lengths

This section elaborates on the process of using CNN for feature extraction from CDR3s. Initially, the CDR3s are embedded using a pre-trained protein-BERT model to generate an embedded sequence matrix. Subsequently, this matrix is treated as the input for the CNN framework, resembling a 2D image analysis. To maintain consistency in input feature dimensions, all sequences are zero-padded to align with the length of the longest sequence, set to 24. The CNN architecture consists of convolutional layers, pooling layers, and linear layers. Various convolutional filters of different sizes are employed to extract short peptide motifs from the input TCR matrix. Finally, 1-max pooling is applied to each convolutional feature, and the results are concatenated.

In our model, we utilize CNN to extract important features from CDR3. By using convolutional kernels of sizes 2, 3, and 4, we can capture information from sequences of varying lengths of 10 to 24 AAs, with sequences of 12 to 16 AAs being the most common. Research by Ostmeyer et al. indicates that short peptide motifs contribute the most to the antigen-binding specificity of TCRs, thus our convolutional kernel design fully considers the importance of n-mer short peptide motifs in antigen recognition [36, 53]. This design maintains the simplicity and understandability of the model structure while ensuring the relationship between the sizes of adjacent convolutional kernels to extract more comprehensive features. We initially determined the appropriate number of convolutional kernels based on the frequency of different length motifs from sequences of 343 healthy individuals and lung cancer patients [54], ultimately resulting in six convolutional layers corresponding to 3, 2, and 1 convolutional layers. Further details can be found in Table S2.

Taking a sequence of length 10, ‘CASSGTEQFF’, as an example, when the convolutional kernel size is $2\times d$, a convolution operation is performed, which effectively extracts sub-sequences such as ‘CA’, ‘AS’, and ‘SS’. With a stride of 1, this results in nine feature sets. For the sequence of length 10, when using convolutional kernel sizes of $3\times d$ and $4\times d$, we obtain eight and seven feature sets, respectively. An activation function is added after each convolutional layer. Since it is a binary classification problem and we compared the performance of the ReLU and sigmoid activation functions under the same parameters, the results showed that the sigmoid function performs better. Therefore, in this study, we choose the sigmoid function as the activation function, as shown in equation 3.


(3) \begin{equation*} \sigma (x)=\frac{1}{1+{e}^{-x}} \end{equation*}

The max pooling layer is used to compress the feature maps output from the convolutional layers into a vector for further fully connected operations. The purpose of 1-max pooling is to select the maximum value on each channel and combine all the maximum values into a vector. Based on biological significance, 1-max pooling can extract the most significant features from the CDR3 sequence. Therefore, in this study, we choose the 1-max function for pooling. Each sequence undergoes 1-max pooling to reduce the feature sets to one dimension. These feature vectors are then concatenated to obtain 6 × 1 feature vectors. Subsequently, through a single linear layer, predictions are made for each sequence, resulting in cancer probabilities. The fully connected layer L is defined in equation 4 below:


(4) \begin{equation*} {\hat{y}}_i=P\left({y}_i=1\right)=\sigma \left({w}_L^T{p}_i+{b}_L\right) \end{equation*}

Among them, $i=1,2,\cdots, 6$, ${p}_i$ represents the feature vector of TCR after 1-max pooling. ${w}_L$ and ${b}_L$ are the weight and bias, respectively. The activation function normalizes the predicted value to the range of (0,1). If ${\hat{y}}_i>0.5$, it is predicted as a cancer sequence; otherwise, it is predicted as healthy sequence.

The cross-entropy loss function better measures the discrepancy between actual output and expected output in the training process of neural network models, facilitating faster convergence to desirable results. Furthermore, in this study, for the TCR classification problem, considering the characteristics of following a Bernoulli probability distribution, utilizing the cross-entropy loss function can enhance model performance and training efficiency. The specific logarithmic likelihood function is depicted as equation 5.


(5) \begin{equation*} \ln L=-\left[{\hat{y}}_i\ln{\hat{y}}_i+\left(1-{\hat{y}}_i\right)\ln \left(1-{\hat{y}}_i\right)\right] \end{equation*}

To avoid overfitting of the model, we set a dropout rate of 40%. The dropout layer randomly sets a certain percentage of neuron outputs to 0, preventing strong dependencies between neurons and improving the model’s generalization ability.

#### MIL and ensemble learning techniques

Previous studies have often overlooked the intra- and inter-sequence interactions of TCRs, using single TCR sequences to predict the probability of cancer or averaging scores for all sequences. Traditional single-instance learning (SIL) methods using only a single TCR CDR3 sequence may not fully capture an individual’s immune status comprehensively. In contrast, MIL methods allow a sample to include multiple CDR3 sequences as one instance, enabling us to capture more immune information and provide a more comprehensive and accurate feature description. We conducted experiments to validate this, clearly showing that when representing a sample with multiple instances, model performance improves, as demonstrated in Fig. S6.

To address this issue, we introduce the concept of MIL. Within the MIL framework, we consider each biological sample as a ‘bag’ containing multiple TCRs treated as distinct ‘instances’. This approach allows us to extract features and make predictions for each instance, and then combine these predictions to obtain the final cancer score for the entire sample. TCRs within each sample hold significant biological functions, reflecting the status of the host T cell immune system and potentially containing crucial information about tumor progression in the human body. This problem naturally lends itself to the MIL framework, as each sample typically contains numerous T cells with different TCRs [55–58].

In our study, we introduce the concept of MIL, targeting a sample set $S=\left\{{s}_1,{s}_2,\cdots, {s}_n\right\}$, where each sample ${s}_i$ contains $k$ TCRs. Firstly, we utilize BERT to embed represent each TCR sequence, then employ CNN for feature extraction and pooling operations, obtaining the feature representation for each instance. Subsequently, we make predictions for each instance’s features through a linear layer. This step enables us to independently obtain prediction results for each instance.

To further enhance the robustness and generalization capability of our model, we introduced the concept of ensemble learning. Ensemble learning combines the prediction results of multiple models (classifiers) to obtain the final cancer score. In our approach, we utilize multi-layered linear classifiers to aggregate the results of $k$ sequences. Each linear classifier outputs a value for the sample, and then the average of these models’ values is taken as the final cancer score. We use the cross-entropy loss function to train these linear classifiers, as shown specifically in equation 6 and equation 7.

In the experiment, when setting the number of linear layers between 1 and 10, the results shows that the changes in the values of loss, accuracy, and AUC become less pronounced when the number of linear layers exceeds 5. This is because as the number of linear layers increases, the risk of overfitting also increases. Therefore, we selected a 5-layer linear classifier based on the highest AUC on the validation set. Compared to a single linear classifier, employing multiple linear classifiers introduces greater computational costs and complexity. As shown in Table S3, it is evident that the trainable parameters increase slightly, along with a minor increase in training time. However, the model performance improves. When demanding higher performance and when the hardware can accommodate these demands, opting for multiple linear classifiers is viable.


(6) \begin{equation*} \hat{Y}=P\left(Y=1\right)=\sigma \left[\frac{1}{m}\left({w}_{L^{\prime}}^T{\hat{y}}_i+{b}_{L^{\prime }}\right)\right] \end{equation*}

where $m$ represents the number of linear classifiers, while ${w}_{L^{\prime }}$ and ${b}_{L^{\prime }}$ represent the weights and biases of the classifiers, respectively. The obtained prediction values are further normalized to the range of (0,1) through an activation function. If $\hat{Y}>0.5$, the sample is classified as cancerous; otherwise, it is classified as healthy.


(7) \begin{equation*} \ln{L}^{\prime }=-\left[\hat{Y}\ln \hat{Y}+\left(1-\hat{Y}\right)\ln \left(1-\hat{Y}\right)\right] \end{equation*}

The binary classification model in this study is designed based on the aforementioned model and is similar to it. In contrast, the multi-classification model employs ReLU as the activation function and does not normalize the predicted probability values. Instead, it selects the class label with the maximum predicted probability as the prediction result. Apart from this difference, the designs of the two models are almost identical.

### Model training and evaluation

Our experiment is conducted on a workstation running Ubuntu 20.04, equipped with an AMD Ryzen R7 5700X processor, 128GB RAM, and an NVIDIA GeForce RTX 4090 (24GB VRAM), using Python 3.9.7 along with torch 1.12.1 + cu102, numpy 1.7.1, pandas 1.3.4, and scikit-learn 0.24.2. During the single disease binary model (Lung and THCA) training phase, We adopted a random partitioning method and used 6:2:2 partitioning for the training set, verification set and test set. The details of data set partitioning for each model are shown in Table S4. we employed the Adam optimizer with an initial learning rate of 0.001. The cross-entropy loss function was utilized as the objective function for the model, and the weight_decay parameter was set to 0.001 for regularization purposes, aiming to mitigate the risk of overfitting. Dropout (set to 40) technique was also employed for regularization during the model training process, where a random subset of neuron outputs is dropped to reduce model complexity. The early stopping strategy is implemented based on the criterion that the validation loss does not decrease for a consecutive number of epochs (set to 40). Specifically, if the validation loss does not improve over the patience threshold of 40 epochs, we terminate the training process. Please refer to Table S5 for the performance of models with different patience levels. To train the model, we initially opted for a lengthy training period of 1000 epochs, with an initial batch size of 100. Throughout the training process, we adjusted hyperparameters, including batch size and dropout regularization parameter, based on the performance on the validation set, ensuring optimal generalization ability and performance of the model. During the parameter tuning process, we recorded the performance of the binary classification model on THCA cancer test samples for different combinations of convolutional kernel count, dropout rate, and learning rate, as detailed in Table S6.

We evaluate the performance of the binary classification models using metrics such as accuracy, sensitivity (recall), specificity, F1 score, AUC, as well as ROC and PRC curves. In the case of multi-class classification tasks, we also employ a confusion matrix to assess the model’s effectiveness. The selection of these evaluation metrics aims to comprehensively measure the classification performance of the models. The confusion matrix can visually show the performance of the classifier. These metrics provide insight into the model’s performance from different perspectives. The measurement metrics are well-known and have been used in previous studies [59, 60] as shown in the following equations.


(8) \begin{equation*} Accuracy=\frac{TP+ TN}{TP+ TN+ FP+ FN} \end{equation*}

(9) \begin{equation*} Sensitivity=\frac{TP}{TP+ FN} \end{equation*}

(10) \begin{equation*} Specificity=\frac{TN}{TN+ FP} \end{equation*}

(11) \begin{equation*} Precision=\frac{TP}{TP+ FP} \end{equation*}

(12) \begin{equation*} F1\ Score=\frac{2\times Precision\times Recall}{Precision+ Recall} \end{equation*}

Where TP (True Positive) indicates the number of samples correctly predicted as positive (immune status of cancer) by the model, TN (True Negative) indicates the number of samples correctly predicted as negative (healthy immune status) by the model, FP (False Positive) indicates the number of samples incorrectly predicted as positive by the model, and FN (False Negative) indicates the number of samples incorrectly predicted as negative by the model. Accuracy is the proportion of correctly predicted samples among all samples. Sensitivity is the proportion of actual positives that are correctly identified as positive. Specificity is the proportion of actual negatives that are correctly identified as negative. The F1 score is the harmonic mean of precision and recall. In summary, F1 score, ROC curve, and PRC curve better reflect the actual performance of the model. To avoid bias, we used all the aforementioned evaluation metrics to compare the performance of the models.

### The universal cancer detection model

To generalize our model in cancer detection research, we integrated data from s 22 groups of PBMC and TIL samples, and 19 groups of healthy PBMC individuals, comprising 2296 samples including 1132 healthy samples and 1164 cancer patients. The training set, validation set, and test set were divided into an 8:1:1 ratio. During training, we trained the model on the training set and saved the model with the best AUC value on the validation set to ensure the convergence of the model. The hyperparameter settings are the same as those of the single disease binary model. The detailed partition of the dataset is provided in Table S7.

### Immune status measurement

We calculate the health score measuring individual immune status and analyze its relationship with age. As shown in equation 13, we introduce a metric called ‘health score’ to measure an individual’s immune status, with higher scores indicating better immune status. The health score reflects the correlation between an individual’s TCR repertoire and their overall health status. A higher health score suggests a lower likelihood of cancer occurrence and a stronger immune ability. When a person’s health score is below 0.5, it is necessary to pay attention to the health status, and conversely, the higher the score, the healthier the immune status of the individual. Therefore, we can correlate the health score with an individual’s immune status to explore the association of TCRs with the functional status, activity, and immune regulation of the immune system.


(13) \begin{equation*} health\ score=1- final\ cancer\ score \end{equation*}

The final cancer score refers to the probability that the model predicts a cancer-related immune status.

### Multiple classifications model

We used three datasets, including globally top-ranked cancers: lung cancer (PRJNA544699) and breast cancer (PRJNA301507), as well as a dataset of healthy individuals [38]. We tuned the parameters based on the performance metrics of the validation set and the optimized parameters from the previous binary classification model. Except that the number of categories is set to 3, the other hyperparameter settings are the same as those of the single disease binary classification model. The data was divided into an 8:1:1 ratio. The detailed partition of the dataset is provided in Table S8.

### Statistical analysis

This study utilized the Spearman correlation test method to analyze data correlations, with the significance level set at *P* < 0.05. Additionally, the Mann–Whitney U test was used to analyze the differences between the two groups, where the symbols ns indicate *P* > 0.05, * indicate *P* ≤ 0.05, ** indicate *P* ≤ 0.01, and *** indicate *P* ≤ 0.001.

## Results

In this section, we introduce the experimental evaluation of the proposed model in three prediction tasks, binary cancer-related immune status prediction, universal model for cancer detection, and multiple classification of immune status prediction. The experiments were conducted on the datasets in the Datasets section.

### Pretraining improves the prediction of cancer-related immune status

Self-supervised pretraining utilizes a significant amount of unlabeled data to acquire comprehensive and expressive feature representations, leading to enhanced performance in supervised learning tasks. This approach has achieved significant success across various domains, including computer vision, natural language processing, and other related fields. Recent research has indicated potential benefits of self-supervised pretraining for protein-related tasks, such as MHC–peptide interactions [47, 49]. However, the application of self-supervised pretraining in immune status prediction tasks remains relatively unexplored. The purpose of this study is to distinguish the TCR repertoire in the blood of cancer patients and non-cancer controls under different immune status.

Regarding the binary cancer-related immune status prediction models, the THCA [61] or Lung [54] as positive samples, healthy samples as negative samples. In order to understand how the pre-trained protein-BERT contributes to improve the prediction of cancer-related immune status, we compared it with the conventional approach commonly used in previous studies, which involves embedding TCRs using the traditional method based on the physicochemical properties of AA. We conducted experiments on multi-instance learning (MIL), comparing the part where each sequence score has different weights (MIL) with the part where each sequence score has equal weights (Mean), in order to demonstrate the enhancement effect of MIL on prediction results. To investigate the importance of the number of classifiers (single or ensemble), we further compared the model incorporating the concept of ensemble learning with the model that does not incorporate ensemble learning in this study. We examined the predictive accuracy of the following four combinations: (i) AA + Mean ( $A{A}^{M\mathrm{ean}}$ ), (ii) AA + MIL + Single ( $A{A}^{MIL}$ ), (iii) Bert +MIL+ Single (BertSingle), and (iv) Bert +MIL+ Ensemble (BertTCR).

**Figure 2:**
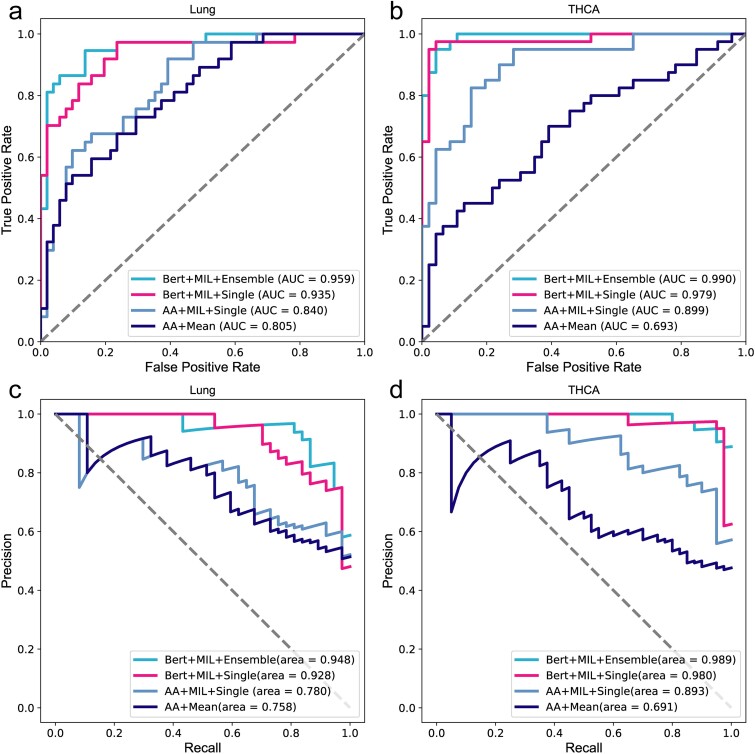
The ROC and PRC curves of models on binary dataset. (a) The ROC curves on lung samples. (b) The ROC curves on THCA samples. (c) The PRC curves on lung samples. (d) The PRC curves on THCA samples.

We employed the four aforementioned combination methods to train THCA and Lung models. For each sample, we selected the top 100 TCRs based on the richness of the processed data. Methods (i) and (ii) encoded the sequences using an AA physicochemical property matrix(20 × 15) [35], while methods (iii) and (iv) utilized protein-BERT for embedding the input AA sequences. CNN was used to extract the features of the four models. Classifiers in methods (ii) and (iii) employed a single linear transformation, whereas method (iv) employed five linear transformations as classifiers. All models were trained until convergence using an identical training set. We repeated this process 50 times and retained the model with the highest AUC value on the validation set. Subsequently, we applied test set to evaluate trained models and summarized accuracy, sensitivity, specificity, F1-score, and AUC metrics in Table 1. Higher values for all these metrics indicate superior performance. The ROC and PRC curves of the test set are shown in Fig. 2.

**Table 1:** The performances of models on THCA and lung cancer test samples.

**Disease**	**Model**	**Accuracy**	**Sensitivity**	**Specificity**	**F1-score**	**AUC**
**Lung**	$A{A}^{M\mathrm{ean}}$	0.477	1	0.1	0.617	0.805
	$A{A}^{MIL}$	0.739	0.676	0.784	0.685	0.84
	BertSingle	0.841	0.73	0.922	0.794	0.935
	BertTCR	0.898	0.784	0.98	0.866	0.959
**THCA**	$A{A}^{M\mathrm{ean}}$	0.5	0.975	0.087	0.645	0.693
	$A{A}^{MIL}$	0.802	0.625	0.957	0.746	0.899
	BertSingle	0.86	0.725	0.978	0.829	0.979
	BertTCR	0.93	0.875	0.978	0.921	0.99

As shown in Fig. 2 and Table 1, BertTCR in this study shows the most advanced performance compared with the other three algorithms. $A{A}^{MIL}$ has better performance evaluation index than $A{A}^{Mean}$, which proves the superiority of the MIL method by considering the intra- and inter-sequence interactions between k sequences in each sample. The performance evaluation metrics of BertSingle consistently outperform those of $A{A}^{MIL}$, with the lung cancer model using BERT exhibiting 12% improvement in AUC. These findings underscore the superiority of pre-trained protein-BERT over traditional encoding methods for AA sequence representation. Additionally, these results highlight the efficacy of employing appropriate feature extraction techniques to enhance model performance. Moreover, BertTCR exhibited superior performance across all evaluation metrics compared to BertSingle, achieving an impressive AUC of 96% for the diagnosis model of Lung cancer and even a remarkable 99% AUC for THCA.

These findings provide compelling evidence that ensemble learning further enhances predictive capability. The outcomes of this experiment offer valuable insights for future advancements in deep neural networks applied to genomic data analysis, emphasizing the criticality of accurately encoding protein and DNA sequences. The research findings indicate that our model focuses on interactions within and between sequences, significantly enhancing the accuracy of cancer-related immune status. Specifically, this model demonstrates notable utility and effectiveness in applications related to thyroid cancer and lung cancer.

### Comparison with existing methods

To validate the robustness of the trained single-disease prediction models for THCA and Lung cancer, we employed THCA [62] and lung cancer [63] external validation set disease type.

**Figure 3:**
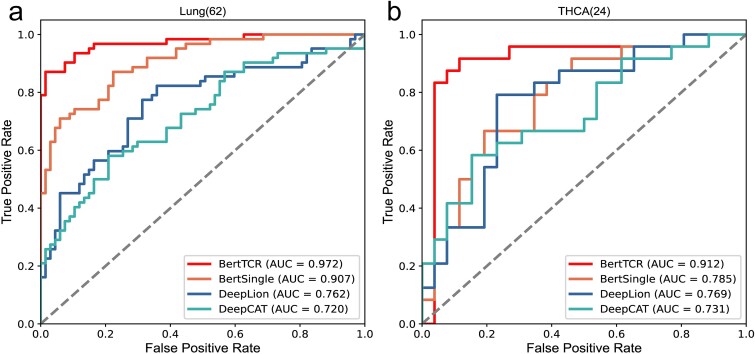
The ROC curves of the external validation set. (a) The ROC curves of lung samples. (b) The ROC curves of THCA samples. The data in parentheses represent the number of samples of the disease.

As mentioned above, we introduce four methods for TCR repertoire classification. Among them, BertTCR uses a classifier based on the pre-trained protein-BERT model and performs well on the NCBI dataset, the detail of usage in Table S1. In comparison, BertSingle only uses a single-layer classifier, and its performance is relatively unstable. DeepLion and DeepCAT methods both use the training models described in Xu and Beshnova’s papers, and encode TCRs using an AA physicochemical property matrix (20 × 15) [35, 38]. DeepLion uses the processed TCRs and extracts features using CNN, with only a single-layer linear transformation used as the classifier. DeepCAT first uses iSMART [20] to perform similarity clustering on the sequences in each sample, and then sets five CNN models to predict according to different AA lengths (length range 12-16), taking the average cancer probability of each sequence as the final result. The performance evaluation results of four different methods on the same external datasets are shown in Table 2. Additionally, Fig. 3 shows the ROC curves for both diseases.

**Table 2:** Comparison of the performance of multiple models.

**Disease**	**Model**	**Accuracy**	**Sensitivity**	**Specificity**	**F1-score**	**AUC**
**Lung**	DeepCAT	0.620	0.871	0.388	0.688	0.720
	DeepLion	0.620	0.887	0.373	0.806	0.762
	BertSingle	0.798	0.871	0.731	0.806	0.907
	BertTCR	**0.899**	**0.952**	**0.851**	**0.901**	**0.972**
**THCA**	DeepCAT	0.600	**0.917**	0.308	0.687	0.731
	DeepLion	0.680	0.583	0.769	0.636	0.769
	BertSingle	0.720	0.583	0.846	0.667	0.785
	BertTCR	**0.900**	0.875	**0.923**	**0.894**	**0.912**

Each boldfaced number indicates the best of the corresponding metric. The BertSingle model was built by us to compare the importance of the key parts of the model.

The results in Fig. 3 and Table 2 clearly demonstrate that BertTCR outperforms the other three methods in all performance evaluation metrics. Combined with the contents in Table 2, the stability of BertTCR prediction model is further illustrated. In the external validation set, the THCA model achieved an AUC value of 91.2%, which is higher than the best-known model’s AUC value of 14.3%, while Lung achieved an AUC value of 97.2% in the external validation set, which is 21% higher than the best model, highlighting its significant feature extraction capability using the pre-trained protein-BERT model. As well as performance enhancements through MIL and ensemble learning in the context of THCA and lung cancer, resulting in outstanding predictive ability.

This validation not only reinforces the robustness and generalization capacity of the BertTCR model but also highlights its potential for clinical applications and transformative impact in cancer-related immune status prediction and cancer diagnosis. These validation results further demonstrate the effectiveness of our model in predicting thyroid cancer and lung cancer.

### Developing a universal model for cancer detection

In order to promote our model in the research of cancer detection, we integrated data from two sources including healthy individuals and various types of cancer, totaling 2296 samples. Among them, there were 1132 healthy individuals and 1164 cancer patients, covering 17 different types of cancer. We processed these data according to the previous method, and used this large-scale processed data to train a universal model capable of cancer detection.

On the test set, we obtained the ROC curve shown in the Fig. S7, with an AUC value of 0.867. Additionally, the model achieved an accuracy of 0.801, sensitivity of 0.792, specificity of 0.811, and F1-score of 0.805. This shows that BertTCR can identify the common features of various cancer sequences, so as to realize the TCR repertoire that can distinguish cancer patients with different immune statuses from healthy people. By assessing an individual’s final cancer score of developing cancer, if the score is high (value greater than 0.5), it should be taken seriously and further detailed medical examination should be sought.

The biological significance of this study lies in the development of an innovative model that utilizes immune system information for cancer detection. This model not only effectively distinguishes between healthy individuals and cancer patients but also identifies common features among different types of cancer. It provides a crucial tool for personalized medicine. Through this approach, we offer new potential for early cancer screening and diagnosis, aiming to assist healthcare professionals in detecting and treating cancer at earlier stages. This has the potential to significantly improve patient survival rates and quality of life.

### External validation of the universal cancer detection model

**Figure 4:**
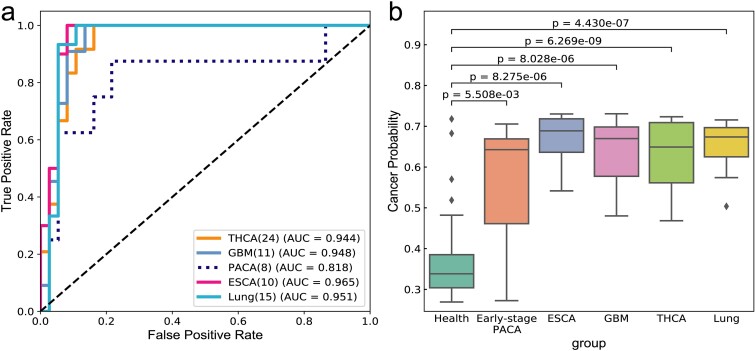
The ROC and PRC curves of the external datasets. (a) The ROC curve of the external datasets. (b) Cancer score for each immune status. The difference between the two groups was analyzed using the man Whitney U test.

The objective of this task was to assess the stability and robustness of the universal model using an external validation dataset. Data from Zenodo database and NCBI database were used, with a cohort of healthy individual and samples of five types of cancer as shown in Table 3. More details of usage are shown in Table S1. The performance results of the model on the external validation dataset are presented in Fig. 4 and Table 3.

**Table 3:** Evaluation of the universal model’s predictions on the external validation dataset.

**Disease**	**Accuracy**	**Sensitivity**	**Specificity**	**F1-score**	**AUC**
THCA (24)	0.885	0.875	0.892	0.857	0.944
GBM (11)	0.896	0.909	0.892	0.800	0.948
PACA (8)	0.844	0.625	0.892	0.588	0.818
ESCA (10)	0.915	1.0	0.892	0.833	0.965
Lung (15)	0.923	1.0	0.892	0.882	0.951

THCA, thyroid cancer; GBM, glioblastoma; PACA, pancreatic cancer and benign cyst; ESCA, esophageal cancer; Lung, lung cancer. The size of the sample is in parentheses.

As can be seen from Fig. 4a, except for the AUC value of 0.818 for PACA, the AUC values of the four cancer types in the general model are relatively high, and the AUC values of THCA, GBM, ESCA and Lung are 0.91, 0.95, 0.97 and 0.95, respectively. As depicted in Fig. 4b, there is a clear difference in the probability of cancer prediction between health and cancer, including early-stage cancer. By correlating these findings with the specificity and sensitivity values outlined in Table 3, the analysis indicates highly accurate predictions for THCA, GBM, ESCA, and Lung. In fact, ESCA and Lung exhibit a sensitivity of 1, whereas THCA and GBM demonstrate sensitivities of 0.88 and 0.91, respectively, indicating overall good performance.

As for PACA, its sensitivity is 0.625. There are two reasons for this: firstly, the small dataset size of only eight samples; more importantly, PACA represents early-stage pancreatic cancer or benign cyst, where the immune system may not be significantly compromised. The TCRs of early-stage pancreatic cancer have a high degree of similarity with those of healthy individuals, making misclassification more likely. This observation indirectly reflects that our model can judge whether the immune status is good or bad based on the cancer score.

These findings underscore the importance of TCR similarity between early-stage pancreatic cancer and healthy status, as well as the challenges of developing and validating a universal cancer detection model with small sample datasets. Despite the sensitivity of early-stage pancreatic cancer and benign cyst is not good enough, our model performs admirably in other types of cancer such as thyroid cancer, glioblastoma, esophageal cancer, and lung cancer, demonstrating high precision in predicting these cancers.

### Measuring immune status with the health score

**Figure 5:**
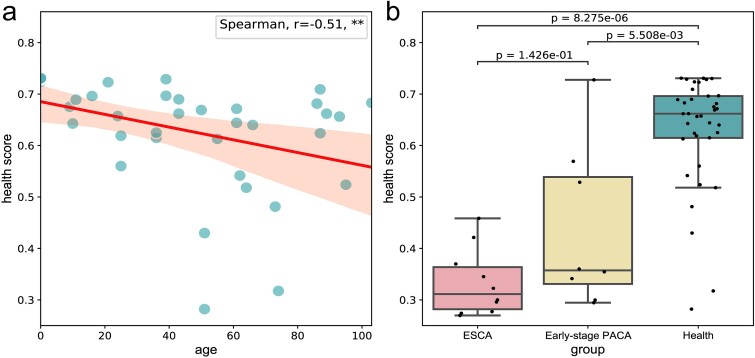
The analysis results about health score. (a) The relationship between age and health scores. Spearman correlation test. Ns: *P* > 0.05, *: *P* < =0.05, **: *P* < =0.01, ***: *P* < = 0.001. (b) The boxplot of health scores for ESCA, early-stage PACA, and healthy individuals. The difference between the two groups was analyzed using the Mann–Whitney U test. Early-stage PACA, early-stage pancreatic cancer or benign cyst; ESCA, esophageal cancer.

Based on the research findings of the previous section, we further analyzed the probability of developing cancer in healthy individuals, and detailed results were shown in Table S9. As shown in equation 13, we introduce a metric called ‘health score’ to measure an individual’s immune status, with higher scores indicating better immune status. We can correlate the health score with an individual’s immune status to explore the association of TCRs with the functional status, activity, and immune regulation of the immune system [64]. This connection contributes to a deeper understanding of the role of the immune system in cancer prevention and maintaining health status. The results of analysis on health score in Fig. 5.

We conducted an analysis on the correlation between health scores and age, calculating the health scores of healthy individuals and utilizing Spearman’s correlation coefficient (r = −0.51, *P* < 0.001). The findings revealed a significant negative correlation between health score and age, as depicted in Fig. 5a. Notably, a declining trend in health scores was observed with increasing age, suggesting a potential decline in immune status and elevated cancer risk. Additionally, among the healthy individuals, four individuals had health scores below 0.5, with ages of 51, 51, 73, and 74, respectively. This finding aligns with a widely held consensus that older adults are more susceptible to cancer. This susceptibility may stem from the natural aging of the immune system, impairing its ability to effectively recognize and eliminate cancerous cells. Therefore, regular monitoring of health metrics is crucial for the elderly population, serving as a critical adjunct for early cancer screening. This proactive approach can facilitate timely interventions and treatments.

Furthermore, in Fig. 5b, among the eight samples of early pancreatic cancer or benign cyst, three samples had health scores exceeding 0.5. This observation holds significant biological implications, suggesting that in the early stages of cancer or benign cysts, the individual’s immune system may maintain a relatively robust functional state. Despite the potential influence of cancer cells on immune responses, the immune system in early-stage cancer may still possess the ability to recognize and eliminate tumor cells, thus providing a crucial window for timely intervention. This finding underscores the critical role of immune surveillance in the cancer development process. Therefore, the magnitude of early cancer health scores falls between those of healthy individuals and those with mid-to-late stage cancer, emphasizing the importance of using such assessment tools in clinical practice. Monitoring immune status through health scores can assist clinicians in promptly identifying high-risk individuals and implementing appropriate preventive measures.

Overall, health scores serves as a proactive indicator for assessing immune status and is poised to play a crucial role in cancer prevention, early detection, and personalized treatment strategies, thereby advancing cancer research in new directions.

### Multiple classifications of immune status prediction

Finally, we conducted a multi-classification task to assess immune status using multiple datasets. This task aims to accurately predict whether the immune status corresponds to a specific type of cancer or a healthy status. To ensure a robust sample size, we selected three datasets (lung cancer, breast cancer, and healthy individuals) with a considerable number of samples. Through training the model, we achieved precise classification of immune status. The performance evaluation metrics are summarized in Table 4, indicating improved performance with higher values across all indicators.

**Table 4:** The performance of multiple classification models.

**Class**	**Accuracy**	**Sensitivity**	**Specificity**	**F1-score**	**AUC**
Health	0.966	1.0	1.0	0.958	1.0
BRCA	0.931	0.933	0.976	0.875	0.977
Lung	0.897	0.750	0.881	0.833	0.942

BRCA, breast cancer; Lung, lung cancer.

**Figure 6:**
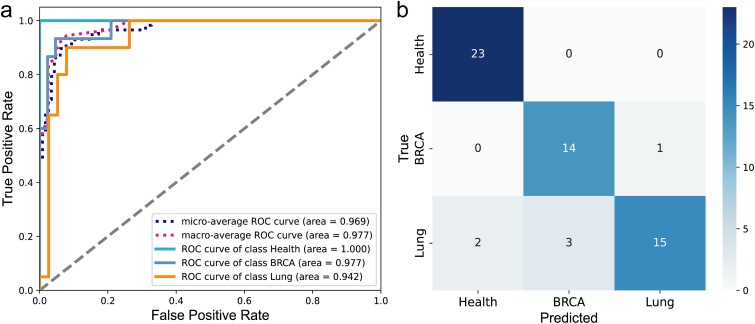
The test results of multiple classification models. (a) Receiver operating characteristic. (b) Confusion matrix.

As shown in Fig. 6 and Table 4, the model performs excellently in the Health category, with accuracy, recall, and specificity all approaching perfection. The F1 score and AUC value are also at a very high level. In the BRCA category, the model exhibits high accuracy and specificity, and the AUC value can reach 0.98. For the Lung category, the model demonstrates high accuracy and specificity, and the AUC value can reach 0.94.

In summary, the model demonstrates excellent performance in a multi-classification task, achieving high accuracy and specificity across different categories. This indicates that the model can effectively distinguish the immune status between healthy individuals and cancer patients, and accurately identify different types of cancer, thereby demonstrating significant clinical utility. Additionally, it also maintains high recall rates and F1 scores in the majority of categories, indicating its strong ability to recognize immune status, which is significant for early cancer detection and the development of personalized treatment plans. Furthermore, the model exhibits a high AUC value, indicating its strong classification ability for different categories.

## Discussion

The results of our study demonstrate the significant advantages of incorporating self-supervised pre-trained protein-BERT, MIL, and ensemble learning in the prediction of cancer-related immune status using TCRs. Our proposed model, BertTCR, exhibited superior performance across various metrics, including accuracy, sensitivity, specificity, F1-score, and AUC, compared to other existed models [26, 34, 36, 38].

Self-supervised pretraining proved to be a crucial step in improving the model’s performance. By leveraging large amounts of unlabeled data, the protein-BERT model developed robust feature representations that enhanced its predictive capabilities. This is evident from the significant improvements in the performance metrics when comparing BertTCR and BertSingle to the conventional AA physicochemical property-based methods, such as DeepCAT [35] and DeepLion [38]. Specifically, the use of protein-BERT embeddings led to an impressive AUC of 99% for THCA and 96% for Lung cancer in the BertTCR model, underscoring the importance of advanced feature extraction techniques in genomic data analysis.

The exceptional performance of the protein-BERT model reveals its potential implications in cancer diagnostics and personalized medicine. The ability to achieve high AUC values for THCA and lung cancer illustrates the model’s capability to effectively identify disease-specific protein sequence patterns. This is crucial for early cancer detection and can significantly affect treatment decisions, paving the way for more tailored therapeutic approaches. Furthermore, advancements in feature extraction and predictive modeling can enhance our understanding of the underlying mechanisms of cancer, thereby leading to improved patient survival rates.

Traditional SIL methods rely on a single CDR3 sequence and often fail to capture all aspects of an individual’s immune status comprehensively. In contrast, MIL allows multiple CDR3 sequences to be treated as a single sample, thereby offering a more comprehensive and accurate representation of immune information [37]. In our study, using the THCA dataset as an example, we conducted a series of experiments to investigate the impact of using different numbers of sequences (such as Top1, Top10, Top30, Top50, Top70, and Top100) on model performance. As shown in Fig. S6 and Table S10, using only one sequence results in significantly lower model performance compared to considering Top100 (k = 100) sequences simultaneously. With an increasing number of sequences, the model’s performance gradually improves. At k = 100, the model achieves 99% performance. To prevent overfitting, we refrain from further increasing the number of sequences at this point.

From a biological perspective, the advantage of this method lies in its ability to provide a more comprehensive reflection of the complexity of the individual immune system. CDR3 sequences represent the specificity of T-cell receptors and are involved in recognizing different antigens. Therefore, immune responses often involve multiple T cells and their corresponding CDR3 sequences. Relying solely on a single sequence may overlook important information and fail to capture the diversity of immune responses. By using MIL to consider multiple sequences, we not only enhance the representation of immune status but also reveal how individuals adapt their immunity in cases of infection, tumor development, or autoimmune diseases.

Ensemble learning significantly enhances model prediction accuracy. By comparing BertTCR (single classifiers) and BertSingle (multiple classifiers), we found that using multiple classifiers notably improves model performance. Ensemble methods achieve higher AUC values and more stable predictions across different datasets, validating their robustness and reliability in clinical applications. Using the THCA dataset as an example, we conducted binary classification experiments for a single disease, comparing the performance, parameter counts, CPU, and memory usage between single and multiple linear classifiers, as detailed in Table S3. Results show that despite slightly increased training and prediction times and parameter counts, multiple linear classifiers substantially enhance classification performance. Our study indicates the need to balance computational costs and complexity while striving for improved classification performance. In scientific research, the demand for higher performance makes the advantages of using multiple linear classifiers more apparent. The improvement in performance is of significant importance in biomedical research, as it enables more effective early detection of cancers and the development of personalized treatment plans.

Our THCA binary classification model was trained with the following computational setup: a GPU with 5.4GB memory, 20GB RAM, 16 CPU cores, and 6GB storage. The training process took ~ 80 min. Directly training the complex models can present practical challenges in typical research and clinical environments. However, these challenges can be effectively addressed by leveraging cloud computing resources or high-performance computing centers at universities and research institutions. To reduce the demand on computational resources, we employed pretrained models for predictions, requiring fewer resources and reducing both time and hardware requirements. Additionally, our study implemented a strategy of saving trained models for direct predictions on test and independent validation sets, achieving testing times as short as ~ 1 second. This approach highlights the feasibility and effectiveness of using pretrained models in resource-constrained environments, ensuring accuracy, reliability, and enhancing the practical applicability of the models.

Our model was designed with scalability in mind. Currently, the model can easily accommodate an increased number of classification categories by adjusting a single parameter. For instance, once we collect a sufficient number (~200 samples per disease) of other types of cancer samples, we can expand the classification categories from the current three classes to 4, 5, or even more categories. Although our model is currently primarily utilized for the screening of breast cancer and lung cancers, the expansion of the dataset with additional samples will enable the model to screen a broader range of cancer types, thereby providing more robust support for early intervention and treatment of diseases.

## Conclusion

In this paper, we propose a deep learning approach named BertTCR for predicting cancer-related immune status. Leveraging a pretrained protein-BERT model, we significantly enhance the embedding of TCRs, highlighting its effectiveness as a feature extraction technique. Furthermore, our CME predictor effectively captures intra- and inter-sequence interactions within TCRs, thereby improving performance. Compared to state-of-the-art methods, our model achieves higher prediction accuracy based on TCRs. The ability to capture these interactions is critical as TCRs exhibit diversity and variability, impacting their binding specificity to antigens and thus influencing immune response efficacy. Thus, BertTCR holds promise for detecting cancer-related immune status from TCR repertoire data. However, the limitation in sample size of cancer cases may affect the performance and generalizability of the model. Future research could further explore BertTCR’s performance across various applications, including larger clinical datasets, optimized model architectures, fine-tune techniques, and its potential applications in immune monitoring and early-stage cancer diagnostics.

**Key Points**

- We have developed a deep learning framework, BertTCR, which address the current limitation of sequence feature extraction, enabling deeper-level feature extraction and contextual information, and explores intra- and inter-sequence interactions of TCRs.
- Compared to three state-of-the-art sequence-based methods, BertTCR achieves a remarkable 21 percentage point improvement in AUC on external validation sets.
- Our model is flexible, allowing cancer-related immune status assessment without external databases and enabling retraining for different external data sources.
- This innovative solution has the potential to detect individuals’ immune status from peripheral blood TCR sequences, offering personalized medical recommendations in healthcare.
- Our multi-class specific cancer diagnostic tool has the potential to expand to more cancer types, enabling non-invasive multi-category cancer detection.

## Supplementary Material

Supp_data_bbae420

## Data Availability

All the raw TCR-seq datasets used in this study are associated with the NCBI (https://www.ncbi.nlm.nih.gov/) and the open-access Zenodo database (https://www.zenodo.org/record/826447#.ZCFI8bJByUk). The data and code are available online at https://github.com/zhangbeibei-min/BertTCR.
