# Supplementary material for: BertTCR: a Bert-based deep learning framework for predicting cancer-related immune status based on T cell receptor repertoire
Source: Brief Bioinform. 2024 Aug 23;25(5):bbae420. doi: 10.1093/bib/bbae420 (PMC11342255; doi:10.1093/bib/bbae420)
Supplement: Supp_data_bbae420 [file supp_data_bbae420.zip › Supplementary_figures_s1-s7.doc]

**Figure S1.** The analysis of CDR3 amino acid sequences in all cancer-related immune status. (a) The distribution of percentages of CDR3s lengths within (1,30). The healthy people (98 samples); The hematological cancer patients (108 samples); The solid cancer patients (141 samples). (b)CDR3 sequences logo in healthy (length=15). (c) CDR3 sequences logo in hematological cancer patients (length=15). (d) CDR3 sequences logo in Solid tumors (length=15).

**Figure S2.** Under different immune status (healthy, hematological cancer or solid cancer), the proportion of remaining sequences after processing with four filtering conditions.

**Figure S3.** Percentage distribution of CDR3 lengths (1-30) after removing sequences under various filtering rules from different immune status. (a) CDR3 length distribution in healthy individuals (98 Samples). (b) CDR3 length distribution in hematological cancer patients (108 Samples) (c) CDR3 length distribution in solid cancer patients (141 Samples).

**Figure S4.** The box plot of TCR sequences Shannon diversity under different immune states after processing with four filtering conditions. (a) The box plot of Shannon diversity in healthy individuals; (b) The box plot of Shannon diversity in hematological cancer patients; (c) The box plot of Shannon diversity in solid cancer patients. (Mann-Whitney U test, ns: p>0.05, No Significant Difference.)

**Figure S5**. ROC and PRC curves of the model (k set to 10). (a) ROC curves. (b) PRC curves. Each sample selects the top ten most abundant.

**Figure S6.** The ROC and PRC curve of the test dataset in binary classification models for THCA exhibit variations due to differences in the number of sequence inputs used.

**Figure S7.** The ROC and PRC curve of the test dataset. (a) The ROC curve of the test dataset. (b) The Confusion matrix of the test dataset.
